# Adsorchromism: Molecular Nanoarchitectonics at 2D Nanosheets—Old Chemistry for Advanced Chromism

**DOI:** 10.1002/advs.202100539

**Published:** 2021-05-16

**Authors:** Miharu Eguchi, Asep Sugih Nugraha, Alan E. Rowan, Joe Shapter, Yusuke Yamauchi

**Affiliations:** ^1^ International Center for Materials Nanoarchitectonics (WPI‐MANA) National Institute for Materials Science 1‐1 Namiki Tsukuba Ibaraki 305‐0044 Japan; ^2^ Australian Institute for Bioengineering and Nanotechnology (AIBN) The University of Queensland Brisbane QLD 4072 Australia; ^3^ JST‐ERATO Yamauchi Materials Space‐Tectonics Project National Institute for Materials Science (NIMS) 1‐1 Namiki Tsukuba Ibaraki 305‐0044 Japan

**Keywords:** adsorchromism, adsorption, chromism, electronic state change

## Abstract

Chromism induced by changes in the electronic states of dye molecules due to surface adsorption is termed “adsorchromism” in this article. These changes of molecular electronic states are induced by protonation, aggregation, intramolecular structural changes, and other processes, depending on the surface environment. Intramolecular structural changes, such as co‐planarization and decreased molecular motion are the most characteristic and interesting behavior of dye molecules at the surfaces, resulting in spectral shift and/or emission enhancement. In this review, adsorchromism at the surfaces of layered materials are summarized since their flexibility of interlayer distance, surface flatness, and transparency is suitable for a detailed observation. By understanding the relationship between adsorchromism and the electronic states of molecules on the surfaces, it will be possible to induce some desired functions which can be realized simply by adsorption, instead of complicated organic syntheses. Thus, adsorchromism has potential applications such as effective solar energy harvesting systems, or biological/chemical sensors to visualize environmental changes.

## Graphical abstract

Chromism induced by changes in the electronic state of dye molecules due to surface adsorption is termed “adsorchromism” in this article. These changes of molecules’ electronic state are depending on the surface environment. Structural changes and decreased molecular mobility are the most characteristic and interesting behavior of dye molecules at the surfaces, resulting in spectral shift and/or emission enhancement.

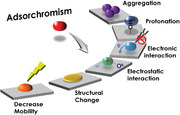

## 1 Introduction

“Chromism” is change in the color of a dye in response to external stimuli and includes vapochromism, solvatochromism, photochromism, thermochromism, piezochromism, electrochromism, mechanochromism, and magnetochromism. The prefix indicates the external stimulus that induces changes in the dye molecules’ electronic states, resulting in the color change. Other color changes used in sensors or indicators are typically referred to not as “chromism” but rather as ion sensors, pH indicators, and redox indicators, which would be ionochromism, acidichromism, and redoxchromism, respectively, if prefixes were used. In this review, the color change caused by adsorption as the external stimulus, thereby changing the molecular electronic states, is termed “adsorchromism” (adsorption + chromism).

Various types of nanoarchitectured adsorbent materials such as porous materials and layered materials have been studied extensively. For example, mesoporous silica materials prepared by the self‐organization of surfactant templates have uniform pore sizes that can adsorb a guest molecule.^[^ ^1^, ^2^ ^]^ Yaghi et al. developed zeolites and MOFs to accommodate guest molecules in their structures.^[^ ^3^, ^4^ ^]^ Generally, these pores are smaller than those of mesoporous materials, and thus these materials are classified as microporous. Compared with the porous materials, layered materials that exfoliate as single layers can accommodate larger molecules on their surfaces. For example, layered perovskite (e.g., K_4_Nb_6_O_17_), and layered aluminosilicate have exchangeable ions at the interlayers that cancel the charge due to isomorphous substitution in the crystal structures, thereby facilitating exfoliation and external adsorption in solvents.^[^ ^5^, ^6^, ^7^ ^]^ Fully exfoliated and dispersed nanosheets provide adsorption sites on both sides of nanosheets a few nm apart.^[^ ^8^ ^]^ Restacking of these hybrid complexes allows the intercalation of adsorbates in the layers, and the flexibility of these layered materials might allow the fabrication of highly designed and controlled reaction fields. Thus, the 2D interlayer area is a unique, extendable adsorption space, allowing the adsorbed molecules to change their conformation. In addition, graphene, graphene oxide, and layered chalcogenides (e.g., MoS_2_) have crystal structures where the bonds between the atoms in a layer are closed, and the layers are bound together mainly by weak van der Waals forces. They are also exfoliated chemically or mechanically to take in molecules.

Layered aluminosilicates are well‐known as traditional clays and are interesting materials for a number of reasons, including their ion exchange capacity, transparency of dispersions and membranes in the visible region, crystallized structure (flat surface at the atomic level), high surface area, acidity, chemical inertness, dispersibility in polar solvents, sufficient thinness for tunneling, safety, abundance, ubiquity, and possibility of artificial synthesis. Ionic molecules adsorb onto layered aluminosilicates primarily through long‐range interaction such as electrostatic forces arising from their ion exchangeable capacity. The protonation of dye molecules by surface acid sites can also result in electrostatic adsorption. Acid sites are often generated through hydrolysis by metal ions but also occur at the edges of aluminosilicate crystals with dangling bonds. Hydrophobic interactions, hydrogen bonds, and interactions between lone pairs of surface oxygen sheets and molecular *π*‐electrons are also involved in adsorption, as well as the London dispersion force and dipole interactions.^[^ ^9^, ^10^ ^]^

**Figure 1:**
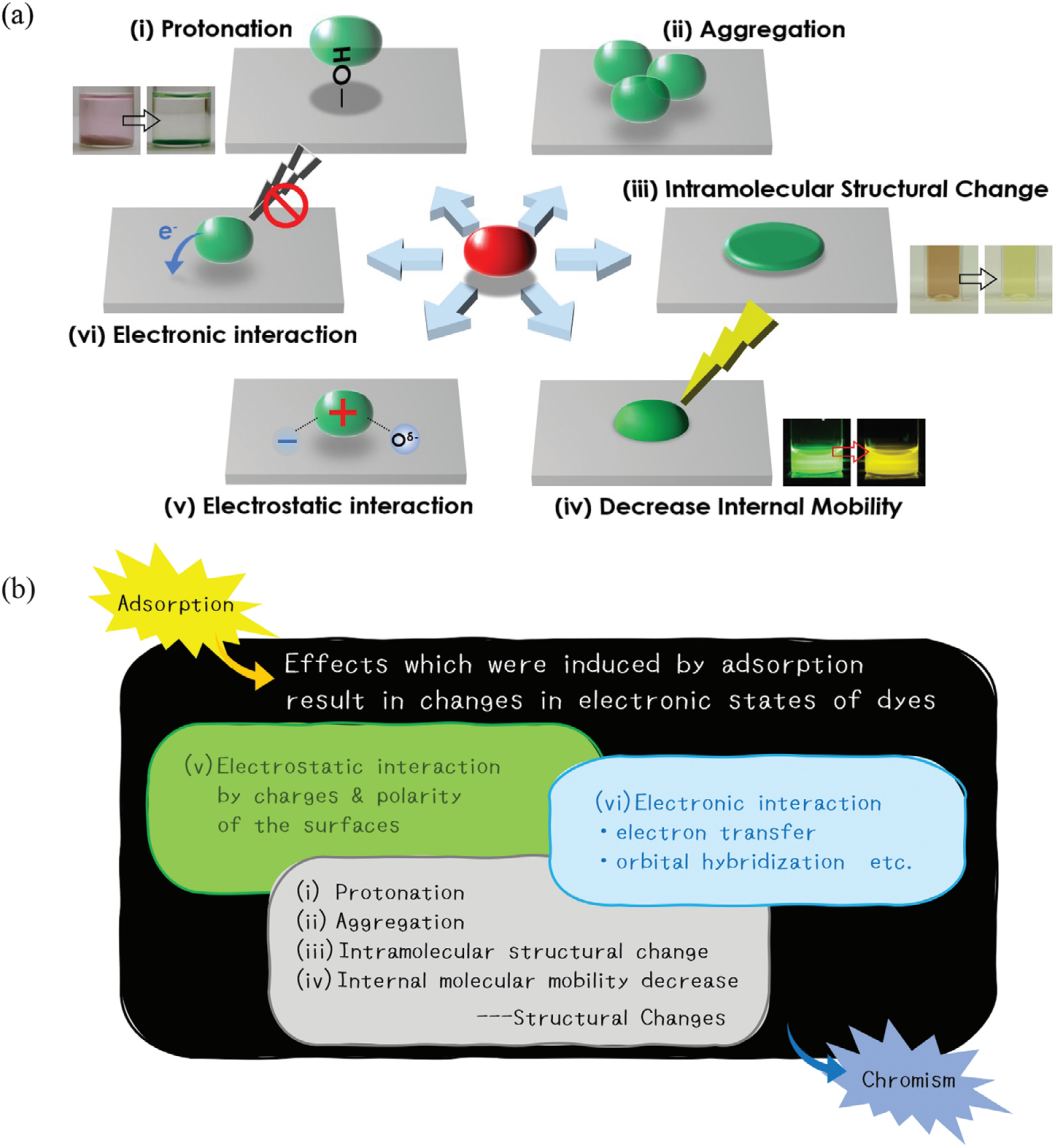
a). Mechanisms of adsorchromism. i) Protonation (photo left: porphyrin on silica, right: porphyrin on aluminosilicate), ii) aggregation, iii) intramolecular structural change (photo left: porphyrin in water, right: porphyrin on aluminosilicate), iv) decrease in internal mobility (photo left: subporphyrin in water, right: subporphyrin on aluminosilicate), v) electrostatic interaction, and vi) electronic interaction (clockwise from top left). b) i–vi)Multiple causes attribute adsorchromism simultaneously.

Interestingly, the photochemical properties of adsorbed molecules can change due to changes in their electronic state on a nanosheet surface. These changes can be induced by i) protonation, ii) aggregation as 2^nd^‐order reactions. As 1^st^‐order reactions, there are iii) intramolecular structural change, iv) a decrease in internal molecular mobility triggered by adsorption, and more fundamentally, v) electrostatic interaction (induced by surface charges and/or polarities), and vi) electronic interaction (electron transfer and orbital hybridization) (**Figure** **1**a). Chromism due to i) protonation can be classified as pH indicator (acidichromism) but the other causes are peculiar to surface adsorption. Furthermore, adsorchromism is always attributed to the simultaneous effects of several of the above triggers (Figure 1b). For example, adsorchromism accompanied by aggregation (ii) is also affected by structural change (iii) and electrostatic interaction (v) at least. For instance, studying adsorchromism due to structural changes with molecules which are unlikely subjected to protonation or aggregation should therefore be informative. Adsorchromism due exclusively to such as surface polarity is difficult to extract and has not previously been studied in detail.

Here, we focus on “adsorchromism” resulting from dye molecules adsorption on 2D nanosheets. We review the history of molecular adsorption, recent advances in understanding the structural and electronic states of nanosheets, their attributes, and effects for tuning colors, and conclude by reexamining interesting perspectives and potential practical applications.

## 2 Adsorchromism by Protonation

Protonation on surfaces is one of the most common causes of color changes by dye molecules associated with adsorption given that layered nanosheets often contain Brønsted acid and Lewis acid sites. In layered aluminosilicates, the Brønsted acid is typically an oxonium ion induced from water due to polarization by the exchangeable cation.^[^ ^11^, ^12^, ^13^, ^14^, ^15^, ^16^ ^]^ The protonation of a dye molecule is dependent on the metal ion hydrolysis potential toward surface‐adsorbed water. For example, the order of acidity on a clay surface is Al^3+^ = Mg^2+^ > Ca^2+^ > Li ^+^> Na^+^ > K^+^ (at a relative humidity of 20%) and Fe^3+^ > VO^2+^ > Mg^2+^ > Na^+^ for montmorillonite.^[^ ^17^, ^18^ ^]^ The Lewis acid sites are generated at the edges of oxides crystals due to bonding deficiencies of Si^4+^ or Al^3+^ to oxygen atoms.

Methylene blue (MB^+^) is a mono‐cationic dye used as a redox indicator (p*K*a: 3.1). The oxidized form is blue and has absorption bands at 665 nm (0–0 vibronic transition) and 605 nm (0–1 vibronic transition).^[^ ^19^ ^]^ The adsorption behavior of MB^+^ onto surfaces supporting protonation and aggregation is well studied.^[^ ^20^, ^21^ ^]^ In 1997, Bujdak et al. found that aggregation (*λ*_max_: 573 nm) was suppressed on the surface of Li^+^‐montmorillonite with a low cation exchange capacity (CEC), resulting in larger distances between MB^+^ molecules. Consequently, the monomeric (*λ*_max_: 675 nm) and protonated forms (*λ*_max_: 775 nm) of MB^+^ (prepared in distilled water) tend to be observed on the surface of Li^+^‐montmorillonite, and protonation occurs due to the hydrolysis of exchangeable cations.^[^ ^22^ ^]^

Non‐ionic, basic molecules also adsorb onto layered aluminosilicates. Heller‐Kallai et al. investigated cyclohexyl diamine (p*K*a: 10.8), ethylenediamine (p*K*a: 9.7), aniline (p*K*a: 4.6), and histidine (p*K*a: 1.8) with Al^3+^‐montmorillonite and Ca^2+^‐montmorillonite dispersed in distilled water.^[^ ^23^, ^24^ ^]^ Adsorption was not stoichiometric, indicating that protonation by adsorbed water hydrolyzed by exchangeable cations, rather than cation exchange, was a major process for adsorption. All four amines (including histidine) adsorbed to both montmorillonite and thus the Lewis acid strength (*H*_0_) of Al^3+^‐ and Ca^2+^‐ montmorillonite is less than 1.8, in agreement with a previously reported *H*_0_ value.^[^ ^25^ ^]^ The amount of adsorbed amine on Al^3+^‐montmorillonite was larger than on Ca^2+^‐montmorillonite, consistent with the order of strength of the metal ions for hydrolysis given above.

In 2018, Nakazato et al. confirmed the adsorption of 4,4′‐bipyridine (bpy; p*K*a_1_: 4.8, p*K*a_2_: 3.2) to Na^+^‐montmorillonite in ion‐exchanged water.^[^ ^26^ ^]^ The equilibrium constant and saturated adsorption amount were calculated to be 4.7 L m^−2^ (=2.2 × 10^6^ L mol^−1^) and 3.2 × 10^−2^ molecules nm^−2^ (corresponding to 55% vs CEC). These values suggest that adsorption is due to protonation by adsorbed water hydrolyzed by the exchangeable cation. The *H*_0_ of Na^+^‐montmorillonite is less than 3.2. These molecules are not dyes but their adsorption properties are informative when considering the adsorption behavior of nonionic dye molecules.

Adsorption due to protonation is observed for non‐ionic porphyrin (tetraphenyl porphyrin, TPP, p*K*a: 3.1) with Na^+^‐saponite.^[^ ^27^ ^]^ The experiments were conducted in organic solvents because TPP is water‐insoluble. TPP and saponite have low affinity and thus solvents with even lower affinity for saponite were required for TPP to approach the saponite surface. TPP then donates a lone pair of electrons from the pyrrole nitrogen to a Lewis acid site generated at the edge of the saponite crystal due to bonding deficiencies of the metal ion, followed by protonation and a color change to green (Figure 1a(i)). The *H*_0_ on saponite was evaluated to be between 0.8 and 1.5 using several pH indicators. Hence, molecules with p*K*a values in this range have the potential to adsorb onto saponite.

## 3 Adsorchromism by Intermolecular Interaction

Aggregation is a common intermolecular structural change in dye molecules on layered surfaces. Aggregation induces spectral changes because molecules are close enough to interact with each other electronically. Based on the exciton theory proposed by Kasha, spectral changes can be classified into three patterns: i) sharper bands at lower energies compared with the monomer, ii) broader band at higher energies than that of monomers, and iii) split bands. Each pattern is assigned as J‐aggregates (head‐to‐tail‐association), H‐aggregates (sandwich‐type assemblies), and aggregation with crossed configuration, respectively. The mechanisms underlying these aggregations and examples of aggregation on the surfaces of layered aluminosilicates are summarized well in articles by Bujdak.^[^ ^28^ ^,29^^]^

MB^+^ and rhodamine 6G (R6G^+^) likely form aggregates in water. Generally, molecular aggregation tends to occur when the molecules are planar chromophores whose transition moments can couple. In contrast, aryl substituents tilted to the chromophore plane can prevent aggregation, explaining why rhodamines aggregate more slowly compared with MB^+^.^[^ ^29^ ^]^ Their aggregation behavior has been intensely studied also on the surface of layered aluminosilicates. According to O'Konski et al., MB^+^ (monomer; *λ*_max_ = 664 nm in water) showed a 6 nm redshift by monomeric adsorption to Na^+^‐montmorillonite and formed J‐ and H‐aggregates with peak positions at 762 and 579 nm, respectively.^[^ ^30^ ^]^ Bujdák et al. determined the orientational angles of the monomer and H‐dimer to be 22° and 28° with respect to the surface.^[^ ^31^ ^]^ Arbeloa et al. reported the monomeric adsorption of R6G^+^ (monomer; *λ*_max_ = 526 nm in water) on the surface of laponite when the loading level was 0.1% versus CEC (peak position: 528 nm), whereas between 1% and 25% versus CEC, J‐ (*λ*_max_ = 545 nm, orientational angle; 29° toward the surface) and H‐aggregates (*λ*_max_ = 503 nm, orientational angle; 42° toward the surface) were observed.^[^ ^32^ ^]^ Higher‐order aggregates (*λ*_max_ = 468 nm) were observed above 40% versus CEC. In both cases, monomeric adsorption does not induce large spectral shifts, due to the tilted orientation. The tilted orientation accelerates aggregation, which is entropically more stable.

Fluorescein has a structure similar to that of rhodamine but is anionic (*λ*_max_ = 495 nm in water). Fluorescein was introduced into the interlayer space of layered double hydroxide ([Mg_3_Al(OH)_8_](CO_3_^2−^)∙*n*H_2_O) with an anion‐exchange capacity (AEC) of 3.25 meq g^−1^. Up to 0.1% fluorescein versus ACE result in monomeric adsorption without noticeable spectral change. Further increase in the loading level results in H‐aggregates appearing at 450 nm, and J‐aggregates were observed at 530 nm up to 50% versus AEC.^[^ ^33^, ^34^ ^]^ As with MB^+^ and R6G^+^, fluorescein is assumed to be tilted in the interlayer space.

## 4 Adsorchromism by Intramolecular Structural Change

Adsorchromism due mainly to intramolecular structural changes is studied using dye molecules that are unlikely to be protonated and/or aggregated. For example, meso‐tetra(4‐*N*‐methylpyridyl)porphine (TMPyP^4+^) has a relatively low p*K*a (p*K*a_1_ = 0.8, p*K*a_2_ = 2.06) and is unlikely to aggregate because of the electrostatic repulsion due to four cationic sites. Redshifts of 14, 28, and 30 nm were observed upon adsorption onto porous TiO_2_,^[^ ^35^ ^]^ H_2_K_2_Nb_6_O_17_,^[^ ^36^ ^]^ and layered aluminosilicates such as saponite,^[^ ^37^ ^]^ respectively. In all cases, the redshift results from delocalization of the *π*‐electrons. The relatively smaller shift observed on columnar porous TiO_2_ (pore diameter > 2 nm) is due to the tilted adsorption of TMPyP^4+^ toward the surface of pores suggested by the random orientation observed by absorption with polarized light. This tilted orientation is due to the higher density of negative charge of TiO_2_ at pH values above the isosbestic point, and thus this spectral shift is characteristic of adsorption and reflects the environment of the dye molecule. Cationic porphyrin derivatives have been studied to understand the relation between spectral and structural shifts by systematically changing their meso‐substituents.^[^ ^37^ ^]^

The absorbance peak position of the Soret‐band of TMPyP^4+^ in water (421 nm) is redshifted by 31 nm by surface adsorption onto saponite, and then further redshifted 33 nm by intercalation.^[^ ^37^ ^]^ The redshift is synchronized with that of the Q‐band (*λ*_em_ of Qx(0,0), Qx(1,0), Qy(0,0), and Qy(1,0) is 636, 585, 554, and 517, respectively in water, which shifted to 669, 616, 583, and 539 on smectite).^[^ ^38^ ^]^ The spectral changes characteristic of protonation (i.e., enhancement of the Qx (0,0) band) was not observed.^[^ ^39^ ^]^ Furthermore, the Beer–Lambert plot remains linear up to a loading level of 100% versus CEC, showing that aggregation at the surface is not involved. This unique adsorption (high density, not aggregated) can occur because the difference in the average distance between anionic sites at the surface of saponite (1.2 nm) and between cationic sites of the porphyrins (1.1 nm) is sufficiently small to prevent intermolecular interactions leading to the formation of aggregates. Supplementarily, the redshift of absorption due to adsorption is also synchronized with the redshift of fluorescence spectra (*λ*_em_ in water, on saponite, and intercalated is 660, 688, and 715 nm, respectively.).^[^ ^40^, ^41^, ^42^ ^]^

A similar spectral shift was reported for TMPyP^4+^ used as a drug molecule intercalated with DNA in 1983 by Pasternack et al.^[^ ^43^, ^44^ ^]^ Given that drug molecules are typically small, planar, and aromatic, they can slip between the base pairs of double‐stranded nucleic acids without major distortion. However, the intercalation of TMPyP^4+^ with DNA was accompanied by an unexpected large absorption band shift, given that the meso‐substituents are nearly co‐planar to the plane of the porphyrin ring. Pasternack and coworkers found that o‐pyridyl TMPyP^4+^, which has a larger rotational barrier, interacted weakly with the bases of the nucleic acids and did not show a spectral band shift, unlike p‐pyridyl TMPyP^4+^. Thus, they concluded that the redshift of the absorption band indicates the rotation of meso‐substituents toward the porphyrin ring plane, allowing TMPyP^4+^ to slip between base pairs.

Chernia et al. came to a similar conclusion using laponite as an adsorbent in 1990.^[^ ^45^ ^]^ They explained the relation between spectral shift and co‐planarization by adsorption. TMPyP^4+^ adsorbs onto laponite nanosheets by electrostatic interaction. The surface adsorption of TMPyP^4+^ onto laponite resulted in a 30 nm redshift. Another 30 nm of redshift was observed when they were intercalated. Even non‐ionic meso‐tetrapyridylporphine (TPyP) showed a 39 nm of redshift at the interlayer compared with that in solution (416 nm in methylene chloride). However, the interaction for adsorption was much weaker than that of TMPyP^4+^. Furthermore, TMPyP^4+^ and TPyP adsorbed on talc (neutral layered aluminosilicate) by specific weaker interactions. The redshift‐width of TMPyP^4+^ and TPyP on talc was 35 and 15 nm, which was similar to that on laponite. Therefore, co‐planarization of the porphyrin ring and meso‐substituents is not only driven by electrostatic interaction between the cationic sites of the porphyrin molecules and the anionic sites of the surface of the layered aluminosilicates; rather, it is driven by the common‐type adsorption in polar environments, that is, adsorption of the molecules via van der Waals interactions, and *π*‐interactions of the pyridyl with oxygen on the surface of aluminosilicates.

Semi‐empirical quantum chemical calculations showed that the energy minimum of THPyP (a tetravalent cationic analogous structure of TMPyP^4+^, in which methyl groups were substituted by hydrogens) is centered at dihedral angles close to 90°. The angles at the bottom of the energy plot are flat, within ±15°. Furthermore, the four MOs (e_g1_, e_g2_, A_1u_, and A_2u_) of charged THPyP were determined at different dihedral angles by ab initio calculations. These results were unlike the classic porphyrin model because of charge transfer from the porphyrin ring to the meso‐substituents due to rotation of the substituents. Lower e_g1, 2_ molecular orbitals (stabilized LUMO) were observed when the pyridyls were twisted. This effect is also observed when electron‐withdrawing substituents are introduced to the porphyrin ring.^[^ ^46^, ^47^ ^]^

Corrections to the four calculated energy levels provided results compatible with the measured transition energies, with 30° and 46° rotations from the vertical causing a 30 and 60 nm redshift in the Soret band with a 12.6 and 46.0 kJ mol^−1^ increase in ground‐state energy. This result indicates that major structural change induces larger spectral shifts. It should be noted that the calculations were in a vacuum and not accounting for the ionic attraction of THPyP to laponite.

In 2012, Ishida et al. experimentally clarified the mechanism underlying the redshift by comparing the relative adsorption equilibrium constants and spectral Soret band shifts following the adsorption of porphyrins with different distances between cationic charges.^[^ ^48^ ^]^ They observed a positive correlation between these factors and concluded that flattening of the meso‐substituent with respect to the plane of the porphyrin ring gives rise to the observed redshift upon adsorption.

Summarizing the above, adsorption induces co‐planarization of the intermolecular dihedral angles, leading to *π*‐electron delocalization and thus a redshift.

Methyl viologen (MV^2+^) is a dye molecule having a simple structure that results in chromism by co‐planarization, and a 22 nm shift of the absorption band (in aqueous solution, 257 nm; on montmorillonite, 280 nm) is observed. Furthermore, Slade et al. suggested that the pyridinium rings of MV^2+^ co‐planarize on the surface of Na^+^‐montmorillonite, based on an analysis using IR‐attenuated total reflectance (ATR) to determine the angle between the major axis of the molecule through the nitrogen and the planar surface of the ATR substrate.^[^ ^49^ ^]^

Major structural change induces larger spectral change, or viewed from the other direction, minor structural change induces less spectral change. For example, less flexible dye molecules such as o‐TMPyP^4+^, tetra‐cationic phthalocyanine, and 5‐methylphenanthridinium chloride showed redshifts of 6, 12, and 10 nm, respectively on the surface of layered aluminosilicate, which are smaller than those of porphyrins with less steric hindrance.^[^ ^50^, ^51^ ^]^ In these cases, the redshifts may be mainly due to electrostatic interaction induced by surface charges and/or polarities. In a homogenous system, *π*–π* absorption spectra of porphyrins are likely to shift to a longer wavelength in a less polar solvent.^[^ ^52^ ^]^ Thus, the observed redshift suggests that the surface of aluminosilicate is less polar compared with water. Discussion of the contribution of electrostatic and/or polar effects on shift widths requires further investigation of electronic state shifts by adsorption.

**Figure 2:**
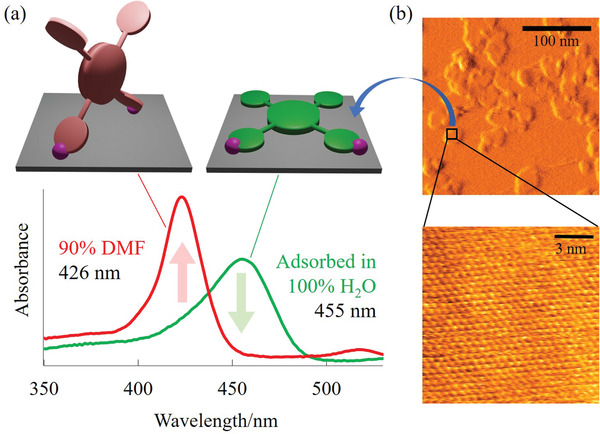
a) Absorption spectral shift by tuning the orientational angle of porphyrin molecules on the surface of layered silicate. (right) The porphyrin ring is parallel to the surface of the aluminosilicate and is co‐planarized with the meso‐substituents, resulting in a *λ*_max_ at a longer wavelength. (left) The porphyrin ring is tilted to the surface of the aluminosilicate and is not co‐planarized with the meso‐substituents, resulting in *λ*_max_ at a shorter wavelength. b) AFM images of the layered aluminosilicate (saponite). (top) Exfoliated single layers. (bottom) Oxygen atom arrangement with sixfold symmetry on the surface of the layered silicate. Images based on ref.^[^ ^40^, ^56^ ^]^

The reverse of “delocalization of *π*‐electron induces redshift” is also true; that is, “localization of *π*‐electrons induces blueshift” For example, ruthenium tris(bipyridine) (Ru(bpy)_3_^2+^), an important photosensitizer, has two absorption peaks at 285 nm (*π*–π* of ligands) and 452 nm (metal‐to‐ligand charge transfer; MLCT; d–π*).^[^ ^53^, ^54^ ^]^ On Na^+^‐montmorillonite, the peaks shifted to 272 and 472 nm. The former peak's blueshift is due to one of the pyridine rings twisting out of plane with respect to the other ring, inducing localization of *π*‐electrons. Furthermore, reversible red‐ and blue‐shift were observed when TMPyP^4+^ changed its orientation on saponite reversibly. In water, TMPyP^4+^ adsorbs onto the surface of saponite (**Figure** **2**b) in a parallel manner,^[^ ^55^ ^]^ with the meso‐substituents co‐planarized (Figure 2a, right). The same adsorption structure was observed in other protic solvents. On the contrary, orientation of TMPyP^4+^ was determined to be tilted in aprotic solvents (Figure 2a, left).^[^ ^56^, ^57^, ^58^ ^]^ Therefore, the parallel orientation was turned out to be induced due to entropy stability to keep the hydrophobic surface area of the porphyrin molecules small. Thus, a reversible spectral shift was accompanied by structural changes by tuning the strength of hydrogen bonding in solvents. This principle was applied to a sensor constructed from films of porphyrin‐saponite hybrid complexes that undergo a color change depending on the surrounding solvent.^[^ ^59^ ^]^ Also, the chromism not accompanied with orientational change but dihedral change was utilized for a humidity sensor.^[^ ^60^ ^]^

**Figure 3:**
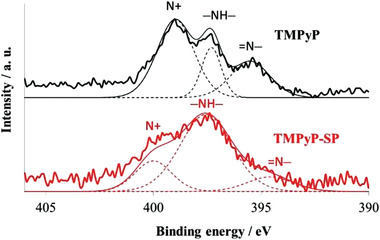
X‐ray photoelectron spectroscopy patterns of N^+^,—NH—, and =N— for TMPyP^4+^ (top) and TMPyP^4+^‐saponite (bottom).

The change in electron distribution resulting from adsorption was investigated using solid‐state ^13^C‐NMR (JEM‐ECA400/JEOL) and X‐ray photoelectron spectroscopy (XPS, ESCA‐3400/Shimadzu, with Mg Κ*α* irradiation) about TMPyP^4+^ and TMPyP^4+^‐saponite (100% vs CEC). The peaks of ^13^C‐NMR assigned to the meso‐ and py(3)‐ position carbon shifted to lower magnetic field by about 5 ppm following adsorption to aluminosilicate, indicating a decrease in electron density at these positions (**Table** **1**, unpublished data).^[^ ^61^ ^]^ XPS measurements yielded spectra (bold lines) that were fitted (solid fine lines) and separated into three peaks (dotted fine lines) using a Lorentzian peak shape (**Figure** **3**, unpublished data). The N 1s peak was fit with three components assigned to the quaternary ammonium cation (N^+^), —NH—, and =N—, in decreasing order of binding energy. The corresponding binding energies were 399.0, 397.3, and 395.5 eV for TMPyP^4+^ (top in Figure 3), and 400.0, 397.6, and 394.6 eV for TMPyP^4+^‐saponite (bottom in Figure 3). A significant shift due to hybridization with saponite was observed for N^+^ and =N—. The shift widths were +1.0, and −0.9 eV, respectively. (Although N^+^ seems to express as if cation is at nitrogen, cation is distributed at hydrogens of methyl substitute for TMPyP^4+^ under vacuum, according to AM1 calculations.^[^ ^50^ ^]^)

Our expectation was that electrons are electrostatically withdrawn by cationic site at methyl‐pyridyl substitute triggered by co‐planarization, judging from the previous report.^[^ ^45^ ^]^ However, the results of NMR and XPS indicate that electrons at methyl‐pyridyl substitutes are decreased with increase of electrons at a part of porphyrin ring by adsorption. Electrostatic neutralization of the cations due to the surface anions could weaken the attraction of electrons in molecules to the cation. Thus, the observed electron distribution fluctuations at N^+^, meso‐ and py (3)‐position carbon are consistent when considered in terms of resonance effect the case without saponite where the cationic charged methyl group behaves as electron‐withdrawing substituent, and the case of with saponite where electrostatically neutralized methyl group behaves as electron‐donating substituent. Judging from almost unchanged chemical shift at py (2)‐ and ipso‐position carbon, and the lowered binding energy of =N—, electrons seem to flow in the porphyrin ring, following co‐planarization by adsorption.

## 5 Adsorchromism by Decrease of Internal Mobility

Generally, the emission from dye molecules is decreased on the surfaces where they are aggregated leading to self‐quenching (as described in the next chapter).^[^ ^34^, ^62^ ^]^ On the contrary, the emission enhancement of dye molecules at layered aluminosilicate surfaces has also been reported. For example, the redox indicator MV^2+^ discussed earlier is nonfluorescent in solution but emits fluorescence via *π*–π* excitation on the surface of Ca^2+^‐montmorillonite and Ca^2+^‐hectorite.^[^ ^63^, ^64^ ^]^ Also, fluorescence (*φ*_F_ = 0.04) from an MV^2+^ derivative with a strained structure was reported by Ledwith et al.^[^ ^65^ ^]^ Based on these previous studies, Detellier et al. suggested that the co‐planarization of MV^2+^ on the surfaces of aluminosilicates increases fluorescence due to a reduction of nonradiative deactivation (the probable main pathway) arising from rotation of the two pyridiniums.

R6G^+^ generally forms aggregates at the surfaces of aluminosilicates (as described in Chapter 3), but Arbeloa et al. showed that at low loading levels (≤0.1% vs CEC) R6G^+^ behaves as a monomer even at the interlayer (film) of Na^+^‐laponite, based on its absorption spectra.^[^ ^66^ ^]^ At the same loading level, they found that the fluorescence quantum yield (*I*_flu_/*A*_exc_; 9.0×10^8^) was increased compared with R6G^+^ in dilute aqueous solution (*I*_flu_/*A*_exc_; 4.9×10^8^). A rigid and constrained environment decreases the internal mobility of aromatic molecules, reducing the internal conversion processes, and enhancing fluorescence quantum yield. In addition, the fluorescence band of monomeric R6G^+^ on laponite (Δ_flu_: 1150 cm^−1^) is wider than that in water (Δ_flu_: 970 cm^−1^) due to changes of the vibronic structure in a rigid environment and distribution of the angle of the phenyl group toward the plane of the main structure. It should be noted, however, that because the shift‐width of R6G^+^ following adsorption is only 1.5 nm (527 → 528.5 nm), the flattening of the dihedral angle is relatively small due to steric hindrance. Furthermore, the observed multi‐exponential decay curves can be attributed to the highly heterogeneous environment. The average lifetime of R6G^+^ on laponite (0.1% vs CEC, 4.2 ns) is slightly longer than that of monomeric R6G^+^ in ethanol (3.9 ns), with monomeric R6G^+^ disappearing at a loading level of 1% versus CEC.

An increase in emission from triplet excites state via MLCT excitation due to adsorption was also observed for Ru(bpy)_3_^2+^ (Its absorbance properties are described previously in this chapter.).^[^ ^67^ ^]^ The maximum phosphorescence band (620 nm in water) of Ru(bpy)_3_^2+^ shifted to 600 and 625 nm on dehydrated and fully hydrated Na^+^‐hectorite, respectively. The phosphorescence quantum yield (0.042) in oxygen‐free water at 20 °C is enhanced two or three times on the surface of hectorite, with increasing the degree of hydration. The enhancement is due to Ru(bpy)_3_^2+^ being covalently hydrated or slightly distorted on the surface of aluminosilicates. Bard et al. determined the emission lifetime of Ru(bpy)_3_^2+^ with and without clay in 1984.^[^ ^68^ ^]^ The lifetime of Ru(bpy)_3_^2+^ on Na^+^‐hectorite (800 ns) was longer than that of Ru(bpy)_3_^2+^ in water (550 ns), consistent with a 30% increase in phosphorescence quantum yield of Ru(bpy)_3_^2+^ on hectorite.

The mechanism of the fluorescence enhancement of cationic dye molecules on the surface of aluminosilicate was studied systematically by Takagi et al.^[^ ^51^, ^69^ ^]^ They estimated causes of fluorescence enhancement as follows. The increase of the radiative deactivation constant (*k*_f_) was attributed to the structure resemblance of the ground and excited states due to immobilization at the surfaces of aluminosilicates, which resulted in the large overlap of the wave functions of the 0–0 transition. Simultaneously, the decrease in *k*_nr_ occurred due to the suppression of the mobilities of rotational groups in the molecules. For example, the pyridinium groups of 1,3,5‐tris[(*N*‐pyridinium)aniline‐4‐yl]benzene are believed to be rotatable and show a 20 nm (296 → 316 nm) redshift following adsorption. The rotation is the main pathway for non‐radiative deactivation, as in the case of MV^2+^.^[^ ^63^ ^]^ The vibrational deactivation path from the singlet excited state on the surface of aluminosilicates is suppressed (*k*_nr_ in water, 0.97 × 10^−9^ s^−1^; *k*_nr_ on clay, 0.19 × 10^−9^ s^−1^), enhancing fluorescence (in water, 0.077 × 10^−9^ s^−1^; on clay, 0.42 × 10^−9^ s^−1^). Other possible factors affecting *k*_nr_ (e.g., the energy gap between the excited and ground states^[^ ^68^ ^]^ and the coupling parameter) are also discussed in this article.

There could be another reason for emission enhancement from *π*–π* transitions, attributed to molecules with rotatable bonds connecting *π*‐electron conjugated groups. These bonds undergo co‐planarization followed by *π*‐electron delocalization upon adsorption, possibly resulting in the *k*_f_ of the molecules increasing due to relatively large overlap between the HOMO and LUMO.

A similar strategy has been used for “aggregation‐induced emission” (AIE) in which emission is enhanced by the suppression of non‐radiative pathways by physical constraint due to intermolecular interactions. Therefore, like AIE, emission enhancement by adsorption has the potential to be developed for practical applications, such as biological probes, optoelectronic systems, and for chemical stimuli response sensings.^[^ ^71^ ^]^

## 6 Adsorchromism by Electronic/Electrostatic Interaction

Electron transfer between a dye and layered material due to adsorption results in chromism, and specifically, emission quenching. Adsorption on a conductive layer such as graphite quenched the fluorescence of Rhodamine B (RhB^+^) and MB^+^ due to electron transfer from the excited dye molecules to graphite.^[^ ^72^ ^]^ On the surface of semi‐conductor, such as layered titanates and niobates, fluorescence quenching of Ru(bpy)_3_^2+^ was confirmed. Furthermore, even layered aluminosilicates, which are usually insulators, induced quenching of photoexcited dye molecules such as Ru(bpy)_3_^2+^ and MV^2+^, because some naturally produced aluminosilicates contain ferric ions (Fe^3+^) in the structures as impurities which accept electrons through electron transfer.^[^ ^64^, ^73^ ^]^ This effect can be used advantageously to enhance the stability of Ru(bpy)_3_^2+^ to prevent degradation.^[^ ^74^ ^]^

Orbital hybridization was confirmed between TMPyP^4+^ and graphite oxide both by a red shift (+20 nm) and a decrease in absorbance following adsorption via hydrogen bond, electrostatic interaction and van der Waals force.^[^ ^75^ ^]^ The orbital hybridization between porphyrins and gold nanoparticles was studied spectroscopically by Sakamoto et al. They concluded that orbital hybridization was depending on the orientation of the porphyrins at the surface of gold nanoparticles, leading the orientation‐dependent carrier dynamics, which determines the performances of organic electronics.^[^ ^76^ ^]^

The mechanism underlying the MLCT redshift (452 to 472 nm) involves destabilization of the ground state of Ru(bpy)_3_^2+^ upon adsorption. XPS measurements conducted by Thomas et al. revealed that Ru 3d_5/2_ binding energy was shifted to higher energies (ruthenium has a 3+ valence on the surface), which would raise the d‐orbital energy in the complex on the surface.^[^ ^53^ ^]^

Dyes such as Fe(tpy)_2_^2+^ polymer (which forms a 1D structure by six‐coordination of 1,4‐bis(2,20:60200‐terpyridine‐40‐yl) benzene at both ends), are used as electrochromic materials and show a small redshift by 4 nm (MLCT: 586 → 590 nm) on the surface of layered aluminosilicate.^[^ ^77^ ^]^ XPS analysis suggested that the binding energy at Fe 2p was decreased on the surface of saponite from 708.5 to 706.3 eV, resulting in an increased HOMO, presumably due to electrostatic neutralization by the anionic sites of aluminosilicate (which is the similar effect observed on TMPyP^4+^/saponite using XPS). This electronic state shift at the center metal by adsorption lowers the operating voltage of electrochromic devices.

The shift direction of XPS was opposite for Ru(bpy)_3_^2+^ adsorption and Fe(tpy)_2_^2+^ polymer adsorption as above. A deeper understanding of these changes of d–π* transition upon adsorption will require further research.

## 7 Conclusion

The significance of dye molecules has been growing for utilization of sun light energy and development of sensors. Adsorchromism is one of the solutions to increase variation of dye molecules’ properties such as absorption wavelength, emission intensity, and electron‐donating potential. Therefore, the induced direct causes of chromism by external stimulus, in this case adsorption, should be understood thoroughly because this will be a useful method to control the dye molecule properties substantially and reversibly. For example, this useful and easier method will replace complicated organic synthesis for molecular modification to some extent. In addition, the union of other chromisms with adsorchromism is essential for more active control of reactivity, as in the case of vapochromism with adsorchromism for humidity sensors,^[^ ^60^ ^]^ photochromism with adsorchromism for artificial muscles,^[^ ^78^ ^]^ and electrochromism with adsorchromism for electrochromic devices.^[^ ^77^ ^]^ From this point of view, there is still room for detailed study, especially about how the surface charge and/or polarity, which has been considered simply as a cause of adsorption, affect to the electronic states in the dye molecules resulting in the color changes. Specifically, spectroscopic analyses such as XPS and solid NMR should be provided more in the future for suggestive information about electron distributions.

**Table 1:** ^13^C NMR assignment of TMPyP^4+^ and TMPyP^4+^/saponite

		**Chemical shift [*σ* ppm^−1^]**	
**Position of carbon**		**TMPyP^4+^**	**TMPyP^4+^‐saponite**
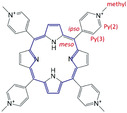	methyl	49.5	49.2
	meso	113.1	118.2
	py(3)	133.5	138.1
	py(2)	144.6	143.6
	ipso	156.5	156.7

## Conflict of Interest

The authors declare no conflict of interest.
